# miRNA Long-Term Response to Early Metabolic Environmental Challenge in Hypothalamic Arcuate Nucleus

**DOI:** 10.3389/fnmol.2018.00090

**Published:** 2018-03-28

**Authors:** Charlotte Benoit, Soraya Doubi-Kadmiri, Xavier Benigni, Delphine Crepin, Laure Riffault, Ghislaine Poizat, Claire-Marie Vacher, Mohammed Taouis, Anne Baroin-Tourancheau, Laurence Amar

**Affiliations:** Centre National de la Recherche Scientifique UMR 9197/Institut de Neurosciences, Université Paris-Sud, Université Paris-Saclay, Orsay, France

**Keywords:** aging, brain, Illumina sequencing, metabolic environment, metabolic programming, miRNA expression, miRNome

## Abstract

Epidemiological reports and studies using rodent models indicate that early exposure to nutrient and/or hormonal challenges can reprogram metabolism at adulthood. Hypothalamic arcuate nucleus (ARC) integrates peripheral and central signals to adequately regulate energy homeostasis. microRNAs (miRNAs) participate in the control of gene expression of large regulatory networks including many signaling pathways involved in epigenetics regulations. Here, we have characterized and compared the miRNA population of ARC of adult male rats continuously exposed to a balanced metabolic environment to the one of adult male rats exposed to an unbalanced high-fat/high-carbohydrate/moderate-protein metabolic environment during the perinatal period and/or at adulthood that consequently displayed hyperinsulinemia and/or hyperleptinemia. We identified more than 400 miRNA species in ARC of adult male rats. By comparing the miRNA content of six biological replicates in each of the four perinatal/adult environments/rat groups, we identified the 10 miRNAs specified by clusters miR-96/182/183, miR-141/200c, and miR-200a/200b/429 as miRNAs of systematic and uncommonly high variation of expression. This uncommon variation of expression may underlie high individual differences in aging disease susceptibilities. By comparing the miRNA content of the adult ARC between the rat groups, we showed that the miRNA population was not affected by the unbalanced adult environment while, in contrast, the expression of 11 miRNAs was repeatedly impacted by the perinatal unbalanced environment. Our data revealed a miRNA response of adult ARC to early metabolic environmental challenge.

## Introduction

The arcuate nucleus (ARC) of the hypothalamus is central for the regulation of the energy balance through adequate neuro/endocrine responses (Timper and Brüning, 2017). The ARC receives information from blood via multiple specialized transporters of nutrients and hormones. Glucose, fatty acids, amino acids, insulin, and ghrelin indicate immediate fuel availability while leptin is indicative of long-term energy store. The ARC also receives information from the cerebrospinal fluid that releases products filtered and/or produced by the choroid plexus. In addition, the ARC receives information from the brainstem that integrates signals from blood through neurons of the postrema area, an area harboring fenestrated capillaries (Rodríguez et al., 2010), and from the gastrointestinal tract through the vagus nerve (Sobrino Crespo et al., 2017). The control of food intake and energy expenditure by the ARC mainly involves two populations of neurons, the orexigenic Agouti-related peptide/Neuropeptide Y (AgRP/NPY) neurons and anorexigenic pro-opiomelanocortin/cocaine-amphetamine-related transcript (POMC/CART) neurons. The ultimate molecular mechanisms whereby the ARC integrates these signals and conveys them to other hypothalamic and non-hypothalamic areas need further investigations, in particular in cases of diabetes and/or obesity.

Epidemiological analyses identified adult unbalanced metabolic environments as one causal factor of metabolic diseases such as obesity and type 2 diabetes. In addition retrospective epidemiological analyses strongly suggested that unbalanced metabolic environments during early-life may potentiate these diseases later at adulthood (Hanson and Gluckman, 2014). Numerous studies conducted in mouse or rat demonstrated, by means of inappropriate diets, the impact of adult and/or perinatal unbalanced metabolic environments on the development of high levels of circulating leptin, insulin, glucose, and/or lipids, contributing then to overweight/obesity and/or insulin resistance/type 2 diabetes at adulthood (Ainge et al., 2011). How unbalanced metabolic environments in early life and/or at adulthood alter mechanisms controlling the energy homeostasis in the central nervous system are currently active research fields.

microRNAs (miRNAs) are small RNAs of 20–24 nucleotides modulating the expression of networks of tens/hundreds of targeted genes (Bartel, 2009; Ha and Kim, 2014). miRNAs and targeted mRNAs form imperfect duplexes that decrease mRNA translation efficiency and/or stability, reducing then the levels of the corresponding proteins. Each miRNA is predicted to control the expression of several hundreds of protein-coding genes because of the short size of miRNA:mRNA heteroduplexes and large amount of putative targets in mRNA transcriptomes. Bioinformatics analyses also predicted that targeted mRNAs might be controlled by multiple miRNAs. In human, mouse and rat genomes, miRNAs are encoded by several hundreds of genes (miR genes). A large fraction of the miR genes is expressed in brain generating highly complex miRNA populations (miRNomes). Different miRNAs have been related to peripheral controls of metabolisms (Vienberg et al., 2017). miRNA involvement in such functions at the ARC level has started to be explored and the possibility that miRNAs of ARC participate in the central control of energy homeostasis has yet to be characterized (Amar et al., 2012; Baroin-Tourancheau et al., 2014; Doubi-Kadmiri et al., 2016). Here, we have identified miRNA expression responses of ARC of adult male rats that had been submitted to perinatal and/or adult metabolic environmental challenges and consequently displayed differences in circulating levels of nutrients and hormones.

## Materials and methods

### Animals

**Figure 1:**
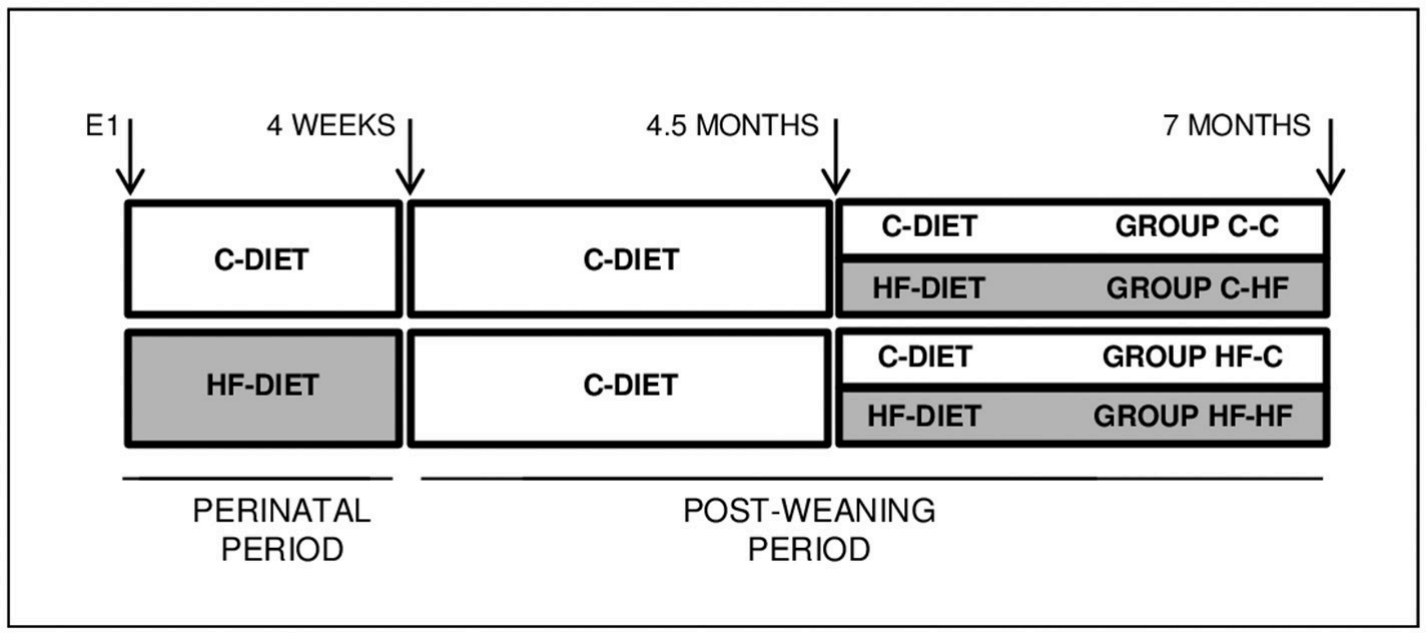
Experimental scheme. Wistar female rats were fed a balanced C-diet (18, 23, and 59% of the energy content derived from lipids, proteins and carbohydrates, respectively) or unbalanced HF-diet (46, 16, and 38% of the energy content derived from lipids, proteins, and carbohydrates, respectively) during the whole perinatal period, from day 1 of gestation up till postnatal week 4. Pups were weaned on the C-diet. Adults of 4.5 months were either maintained on the C-diet or shifted to the HF-diet for 10 weeks. Animals were euthanazied at the age of 7-months after a 14–16-h overnight fasting. Groups were named according to their respective perinatal and adult diets, i.e., C-C, C-HF, HF-C, and HF-HF.

All animals were maintained under a 12/12 Light/Dark cycle (lights on at 8 a.m.) with stable temperature (22 ± 1°C). Virgin females Wistar of 4 weeks and males Wistar of 8 weeks from CERJ Janvier, France, have been habituated to our facilities for 4 weeks before breeding. Standard balanced diet (C-diet) (18, 23, and 59% of the energy content derived from lipids, proteins, and carbohydrates, respectively, Safe, France) and water were provided *ad libitum*. From day 1 of pregnancy and until weaning, dams were either maintained on the standard diet or shifted to an unbalanced diet (HF-diet) (46, 16, and 38% of the energy content derived from lipids, proteins, and carbohydrates, respectively, Safe, France) (Figure 1). At birth, litters were sized to 10 pups to prevent lactation under- or over-nutrition. Upon weaning, animals were fed the C-diet. At the age of 4.5 months, and for the next 10 weeks, they were either maintained under the C-diet or shifted to the HF-diet. At 7 months adults were submitted to an overnight fasting of 14–16-h before being anesthesied with isoflurane, weighted and killed by decapitation. Brains were removed, frozen in isopentane at −40°C for 2 min and stored at −80°C. Bloods were collected in the presence of 50 μl/ml of 160 U/ml heparin. All procedures were conducted according to the guidelines of laboratory animal care and were approved by the local governmental commission for animal research: Ethic Committee for animal experimentation of Paris Center and South # 59 (France), with authorization # 91-467.

### Insulin and leptin measurements

Insulin and leptin plasma levels were measured using Elisa according to the manufacturer's protocol (Millipore). Twenty-five microliters of plasma were used for each point.

### ARC dissection, RNA extraction, and purification of short RNAs

ARC is ventrally adjacent to the 3rd ventricle. A 2–3 mm section was cut from brains, 1 mm rostral to the optic chiasma, and ARC was punched with relation to the ventral part of the 3rd ventricle. ARC-containing punches (ARC punches) were stored individually in Ceramic Bead tubes (Ozyme) at −80°C. The paraventricular nucleus (PVN) that was used as a non-POMC expressing tissue is located dorsally and laterally to the 3rd ventricle. A 1 mm section was cut from brains immediately rostral to the optic chiasma. The PVN was punched with relation to the dorsal part of the 3rd ventricle. PVN-containing punches (PVN punches) were stored as described above.

On purpose, ARC- or PVN-containing Ceramic Bead tubes were added QIAzol lysis reagent (Qiagen) (700 μl) and immediately homogeneized using a PreCellys homogenizer (PreCellys 24/Cryolys) for 20 s at room temperature. After addition of chloroform (150 μl), homogenates were centrifugated 15 min at 13,200 rpm to separate the organic and aqueous phases. The latter was recovered and nucleic acids, precipitated by adding one volume of isopropanol. Pellets were centrifuged, rinsed twice with ethanol 70%, and resuspended in RNase-free H_2_O (20 μl). The yield of total RNA was consistently around 1–2 μg.

**Figure 2:**
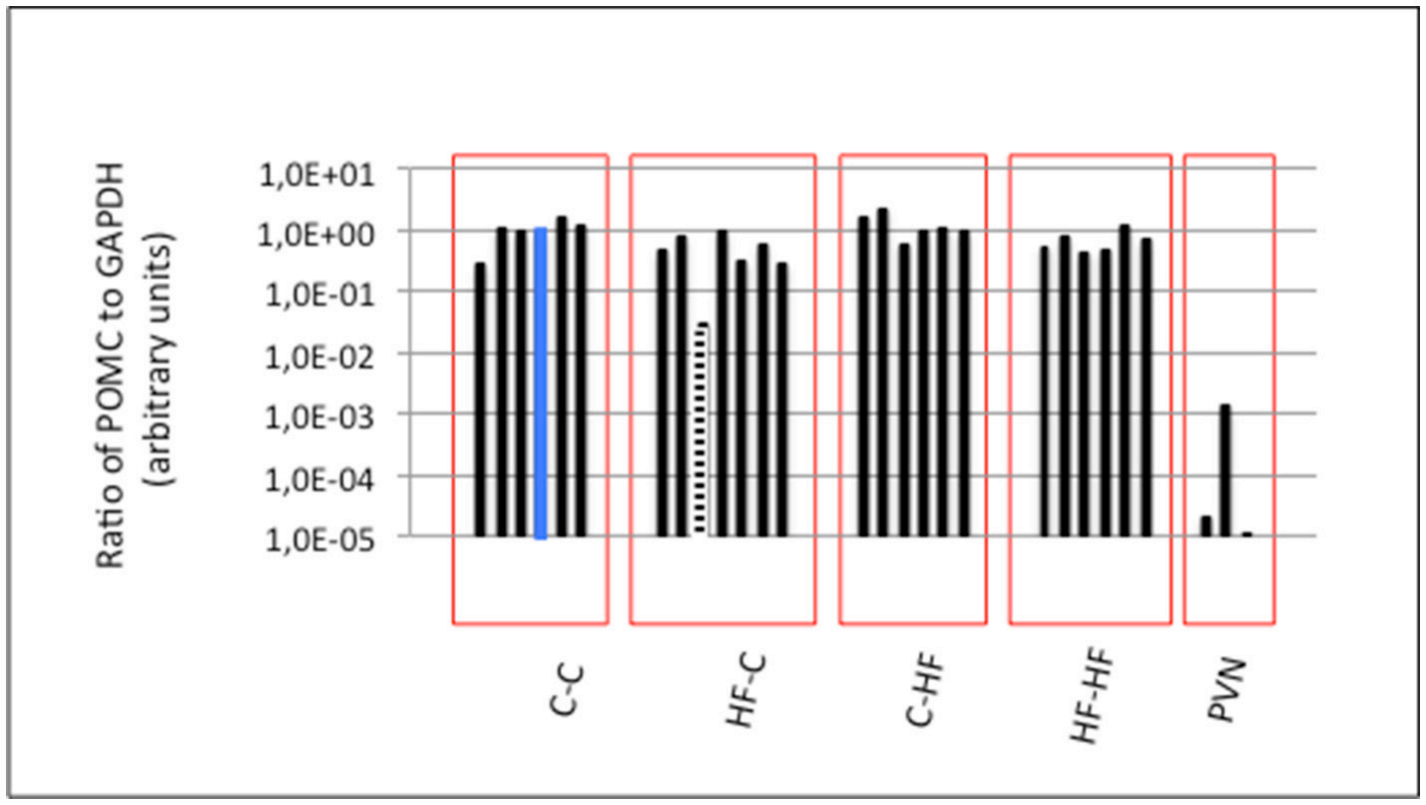
ARC molecular characterization. The ARC harbors a neuronal population specifically expressing POMC transcripts when compared to the surrounding hypothalamic nuclei including the PVN. GAPDH and POMC expression were analyzed by RT-qPCR. POMC expression was normalized to GAPDH expression in each ARC and PVN. Ratio in one ARC of Group C-C (blue bar) was taken as the reference ratio. Ratios are shown using a log_10_ scale. All ARCs displayed POMC to GAPDH ratios at least a hundred-fold higher than those characterizing the PVNs except one ARC of Group HF-C (hatched bar) that was excluded from further analyses. Twenty-four ARCs (six ARCs/group) were conserved for analyses of miRNA populations.

To check the quality of the hypothalamic nucleus dissection, we took advantage of the presence of a neuronal population specifically producing POMC transcripts in the ARC when compared to the surrounding hypothalamic nuclei including the PVN. We determined the level of POMC transcripts relatively to that of Glyceraldehyde 3-phosphate dehydrogenase (GAPDH) transcripts using DNA-free RNAs and RT-qPCRs (Figure 2). Three independent hypothalamic PVNs of adult males were taken as POMC-non-expressing controls. Taking the ratio of POMC to GAPDH transcripts of one ARC of group C-C as the reference ratio, all ARCs but one displayed ratios at least ten-fold higher than ratios characterizing PVNs (low amounts of POMC mRNAs identified in PVNs were due to mRNA axonal transport from ARC to PVN). The low POMC-expressing ARC was discarded and 24 ARCs (6 ARCs/group) were conserved for expression analyses of miRNA populations.

Two-thirds of total RNAs were added an equal volume of formamide, heated at 70°C for 3 min, and loaded on a denaturing urea (8 M) polyacrylamide (17%) gel for size-fractionation. In all cases, the high quality of RNAs was checked by a lack of any smear and the fact that the tRNAs, 5S RNA and 5.8S RNA migrated as discrete bands. Small RNAs of 18–36 nucleotides were eluted from the corresponding slices by overnight incubation in NaCl 0.4 M (0.8 ml) at 4°C under gentle shaking. Eluats were precipitated by adding 2.5 volumes of ethanol in the presence of glycogen (10 ng), rinsed twice with ethanol 70%, and resuspended in RNase-free H_2_O (10 μl).

### cDNA library construction

Individual cDNA libraries were built following an Illumina-like protocol in which 3′- and 5′-adapters were sequentially ligated at the 3′- and 5′-ends of small RNAs, respectively, to allow for their reverse transcription (RT) and amplification by polymerase chain reaction (PCR) as previously described (Doubi-Kadmiri et al., 2016). The 3′-Adaptor was first adenylated by using 25 pmoles of oligonucleotides, 2 × Quick Ligation reaction buffer (New England Biolabs), and 1,600 U of T4 DNA ligase (New England Biolabs), in a volume of 50 μl and by overnight incubation at 37°C. The adenylated and non-adenylated adaptors were size-fractioned on a denaturing urea (8 M) polyacrylamide (20%) gel. The adenylated adaptor was eluted as described above. [0.25 μM] adenylated 3′-adaptor (0.5 μl) was then added to small RNAs (5 μl) in 0.2 ml Thermo-Tubes (Thermo Scientific). To prevent secondary structures, mixes were heated for 3 min at 70°C, then kept on ice while adding [50%] PEG 8000 (2.4 μl), [200 U/μL] truncated T4 RNA ligase 2 (0.4 μl), and 10X truncated T4 RNA ligase 2 buffer (all provided by New England Biolabs) (1.0 μl). Mixes were incubated for 90 min at 25°C. After the addition of [1 μM] 5′-adaptor (0.5 μl) and a new 70°C/ice cycle, mixes were added with [20 U/μl] T4 RNA ligase 1 (0.75 μl) (New England Biolabs) and [10 μM] ATP (1 μl) and incubated for 90 min at 25°C. In a third step, mixes were added with [100 μM] RT-primer (0.5 μl), submitted to a 70°C/ice cycle, then added with [0.1 M] DTT (1 μl), [10 μM each] dNTP (1 μl), 10X buffer (4 μl), and [200 U/μl] of Superscript III reverse transcriptase (0.65 μl) (all from Life Technologies, ThermoFischer Scientific) and incubated for 90 min at 50°C. Finally, mixes were added with [100 μM] 3′-PCR-primer (0.6 μl), [100 μM] 5′-PCR-primer (0.3 μl), and 2X Master Mix Phusion enzyme (15 μl) (New England Biolabs). Reaction mixes were split into two tubes to enhance thermic exchange, denaturated for 1 min at 98°C, and submitted to 16 cycles of 20 s at 98°C, 30 s at 55°C, 25 s at 72°C. PCR products were size-fractionated on a 6% polyacrylamide gel so that ~100-bp cDNAs could be separated from the 75-bp byproducts corresponding to primer dimers. We used a set of 3′-PCR-primers with whole complementarity to the 3′-adaptor but a bulge of two nucleotides at position 22 that were showed not to introduce any bias in control cDNA libraries built from RNAs of one ARC. cDNA libraries built from biological replicates were barcoded with the same 3′-PCR-primer and sequenced in different lanes of an Illumina's genome analyzer GAIIX (Baroin-Tourancheau et al., 2016).

### miRNA expression profiling

Sequencing quality was ascertained using the FASTQC program (http://www.bioinformatics.babraham.ac.uk/projects/fastqc). cDNA libraries were demultiplexed using our scripts on the basis of the first 11 nucleotides of the 3′ adaptor and reads were trimmed from the 3′ adaptor sequence. Sequencing reads <15 nucleotides which could not be mapped on the genome were discarded. Duplicate reads >15 nucleotides were collapsed into unique sequences and analyzed with the sRNAbench tool on the sRNAtoolbox server (http://bioinfo5.ugr.es/srnatoolbox) to build individual miRNA expression profiles (Rueda et al., 2015). This server used the miRBase database version 21 (http://www.mirbase.org) to identify miRNA sequences (Griffiths-Jones et al., 2008). miRNA expression profiles were normalized using the DESeq procedure (Anders and Huber, 2010).

### Statistical measures

Individual group statistics were calculated using Mann and Whitney tests and corrected for multiple testing when necessary according to the false discovery rate method described by Benjamini and Hochberg (1995).

## Results

### Metabolic responses to early and/or adult environmental challenges

All male and female rats have been mated while fed a balanced control diet (C-diet). Then, pregnant females were fed either the same C-diet or shifted to an unbalanced diet rich in fat and carbohydrates and moderate in protein (HF-diet) from the first day of gestation until pup weaning (see Figure 1). Pups were fed the C-diet until the age of 4.5 months. For the next 10 weeks, animals were either maintained under the C-diet or shifted to the HF-diet. Animal groups were named according to their respective perinatal (1st capital letter) and adult (2nd capital letter) diets. For each group, i.e., C-C, C-HF, HF-C, and HF-HF, we used six male rats obtained from three different litters in order to avoid breeding and/or housing bias.

**Figure 3:**
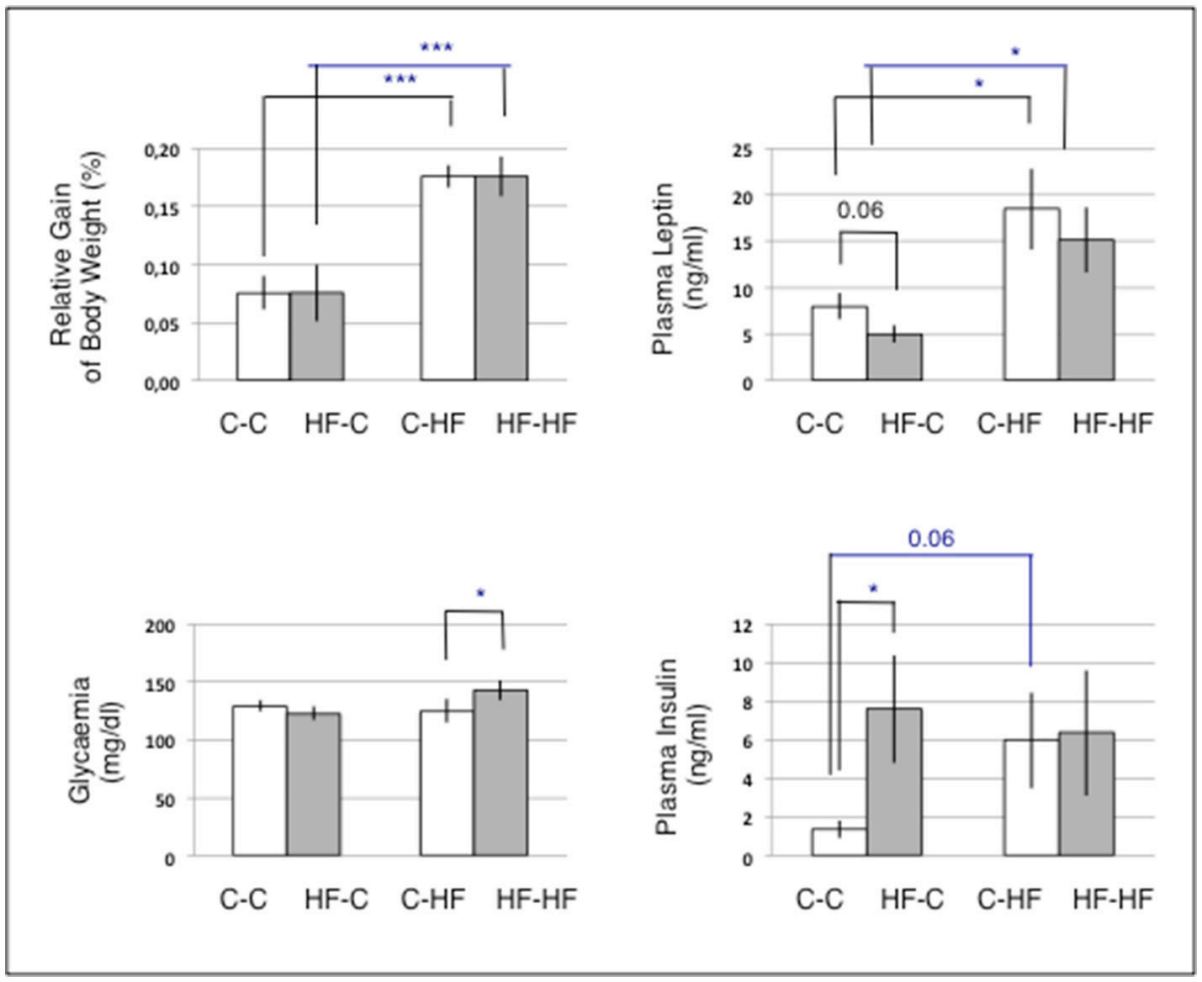
Body weight gains and plasma parameters. For each animal, the gain of body weight was calculated relatively to the body weight at the age of 4.5 months at which animals had been either maintained on the C-diet or shifted to the HF-diet. *n* = 6/group. Statistics were performed using Mann and Whitney tests. Data are given as mean ± SEM. ^*^p < 0.05; ^***^p < 0.001. Challenging the perinatal and/or adult diet resulted in differences in circulating nutrients and hormones involved in the lipid and carbohydrate metabolisms.

Animal groups fed the C-diet at adulthood (C-C and HF-C) displayed similar body weight gains (*p* = 0.31) and slight difference in plasma leptin levels (C-C: 8 ± 1 ng/ml; HF-C: 5 ± 1 ng/ml, *p* = 0.06) (Figure 3). Both groups displayed similar levels of glycaemia (*p* = 0.36) but significant difference in plasma insulin levels (C-C: 1.4 ± 0.5 ng/ml; HF-C: 7.6 ± 2.8 ng/ml, *p* = 0.03).

Animal groups fed the HF-diet at adulthood (C-HF and HF-HF) displayed similar body weight gains (*p* = 0.32) and plasma leptin levels (*p* = 0.29). Those body weight gains and plasma leptin levels were significantly increased when compared to those of groups fed the C-diet (*p* < 1.0E-3 and 0.03, respectively). Plasma insulin levels were similar in groups C-HF and HF-HF (*p* = 0.39). This contrasted with a significant difference in glycaemia (C-HF: 123 ± 5.9 ng/ml; HF-HF: 143 ± 8.7 ng/ml; *p* = 0.03). Plasma insulin levels of groups C-HF and HF-HF were similar to that of group HF-C.

Challenging the perinatal and/or adult diet produced, as expected, four groups of adult rats differing in circulating nutrients and hormones involved in lipid and carbohydrate metabolisms.

### miRNA expression displays high intra-group homogeneity

**Figure 4:**
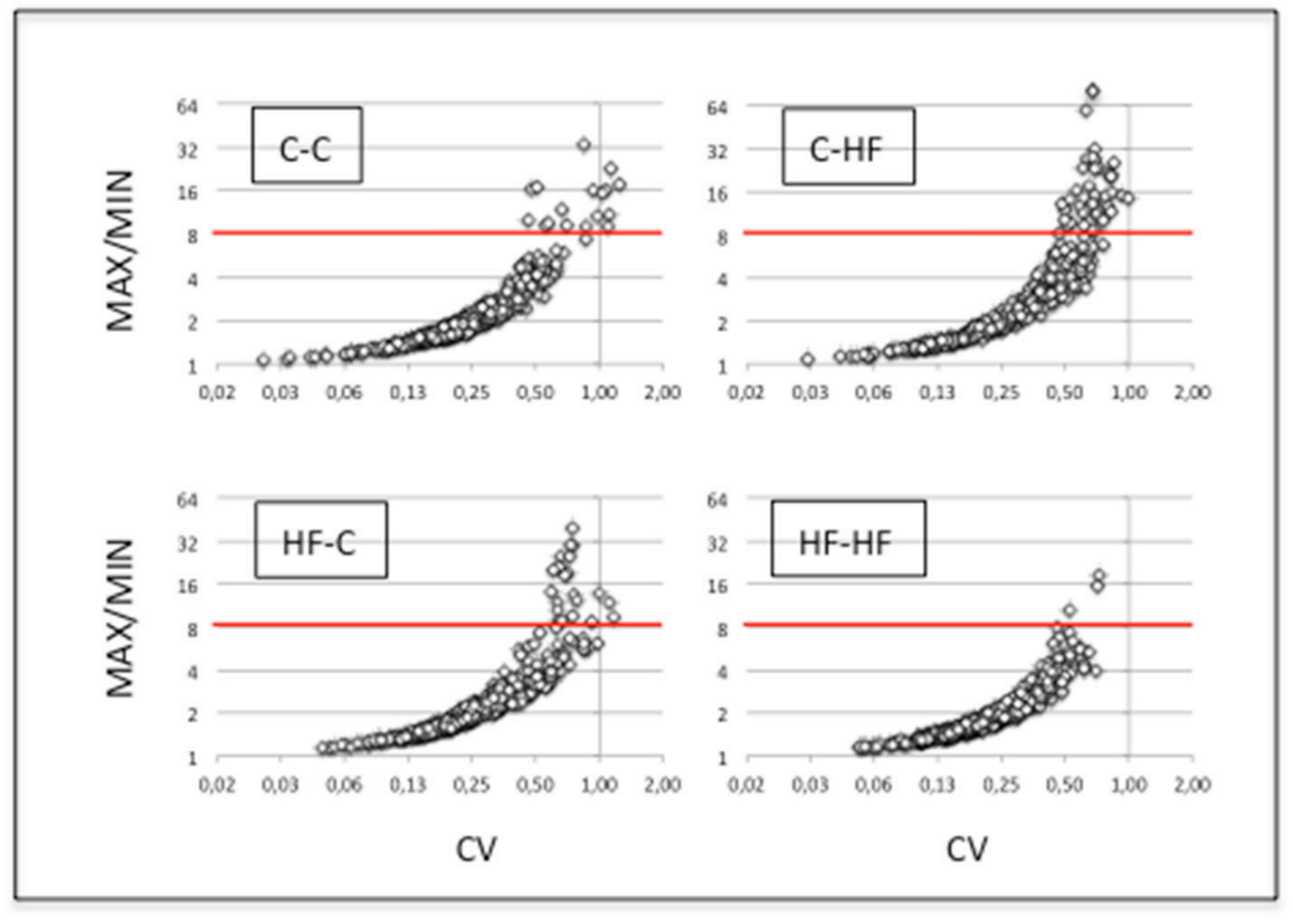
miRNA expression displays high intra-group homogeneity. We characterized complex populations of some 400 miRNAs. In each group and for each miRNA, we analyzed the variation of expression between the six profiles by calculating the maximal to minimal ratio (MAX/MIN) and the standard deviation to mean ratio (coefficient of variation; CV). For each group, scatter plots of MAX/MINs against CVs are drawn. Note that X- and Y-axes used a log_2_ scale. Red lines identify the threshold value of mean + 2 STD in group C-C (i.e., 8.3). More than 94% of the miRNAs displayed close MAX/MINs and/or CVs in each group indicative of extensive intra-group homogeneity of miRNA expression.

Molecular characterization of ARCs was performed in order to check the quality of the dissection (see Figure 2) and small RNAs (16–36 nucleotides) were used for the construction of individual cDNA libraries (Supplemental Table S1) (see section Materials and Methods). A miRNA expression profile was built for each ARC (Supplemental Table S2) (see section Materials and Methods). In each group, the six profiles were then normalized (Supplemental Table S3). Using a mean normalized expression threshold of 10 reads, we characterized complex populations of 380–446 miRNAs in groups C-C, C-HF, HF-C, and HF-HF. In each group we analyzed miRNA expression variation between the six profiles. For each miRNA, we plotted the maximal to minimal ratio (MAX/MIN) against the standard deviation to mean ratio (coefficient of variation; CV) (Table 1, Figure 4).

**Table 1:** miRNA expression profiles.

		**C-C**	**C-HF**	**HF-C**	**HF-HF**
**miRNAs (N)**		**417**	**380**	**446**	**440**
MAX/MIN	MEAN	2.6	3.5	3.2	2.2
	STD	2.9	6.1	4.2	1.6
	MEAN + 2 STD	8.3	15.7	11.6	5.5
	miRNAs with MAX/MIN >8.3 (N)	**15**	**21**	**25**	**6**
	miRNAs with MAX/MIN >8.3 (%)	**3.6**	**5.5**	**5.6**	**1.4**
CV	MEAN	0.28	0.29	0.32	0.24
	STD	0.18	0.16	0.20	0.13

*Statistics. Expression profiles of male adult ARCs display complex populations of 380–446 miRNAs. We analyzed the variation of expression of each miRNA between the six profiles of groups C-C, C-HF, HF-C, or HF-HF by calculating its maximal to minimal ratio (MAX/MIN) and standard deviation to mean ratio (coefficient of variation; CV). Only a minor fraction of the miRNA population of each group displays MAX/MIN values higher than the value of 8.3 that corresponds to mean + 2 STD in group C-C: 1.1, 3.6, 5.5, and 5.6% in groups HF-HF, C-C, C-HF, and HF-C, respectively. Extensive intra-group homogeneity of miRNA expression in all groups is an important feature for sound analyses of inter-group differential expression*.

**Figure 5:**
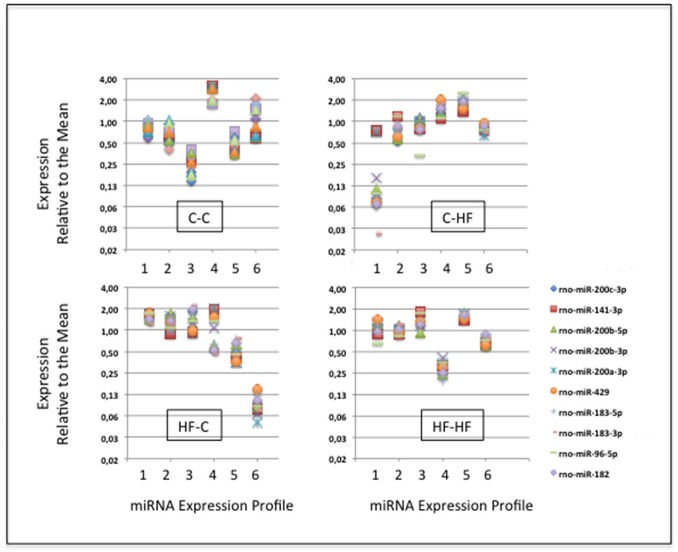
The specific case of the miRNAs specified by clusters miR-96/182/183, miR-141/200c, and miR-200a/200b/429. Ten miRNAs are produced by clusters miR-96/182/183, miR-141/200c, and miR-200a/200b/429. In each group, in each of the six profiles, and for each miRNA, we calculated the expression relative to the mean by dividing individual miRNA read counts by the mean miRNA read count. The expression of the 10 miRNAs co-varies in each profile.

In group C-C taken as the reference, a large fraction of the miRNAs displayed close MAX/MINs (mean ± STD-values = 2.6 ± 2.9) and CVs (0.28 ± 0.18). Indeed more than 96% of the miRNAs had MAX/MINs lower than the value of mean + 2 STD (i.e., 8.3) taken as a threshold value for identifying hypervariable miRNAs (see Table 1). Among the 15 (3.6%) miRNAs displaying MAX/MINs larger than this value, 8 were produced from three miR gene clusters, namely the miR-96/182/183 cluster and the two evolutionary-related clusters miR-141/200c and miR-200a/200b/429 (Table 2). Noticeably these 8 miRNAs as well as the other 2 miRNAs produced from the three miR gene clusters displayed co-variation of expression in each profile (Figure 5).

**Table 2:** miRNAs specified by clusters miR-96/182/183, miR-141/200c, and miR-200a/200b/429 display hypervariable expression.

	**MAX/MIN**			
**miRNA**	**C-C**	**C-HF**	**HF-C**	**HF-HF**
miR-200a-3p	5.9	2.7	**39.3**	4.9
miR-200a-5p				
miR-200b-3p	**16.0**	**12.1**	**24.8**	**4.0**
miR-200b-5p	**22.6**	**17.5**	**20.8**	**7.3**
miR-200c-3p	**11.1**	**24.4**	**19.1**	**6.1**
miR-200c-5p				
miR-182	**10.8**	**27.2**	**18.7**	**6.7**
miR-183-3p	**15.5**	**70.1**	**29.4**	**5.4**
miR-183-5p	**9.1**	**31.7**	**14.1**	**7.8**
miR-141-3p	4.5	1.9	**25.0**	**5.6**
miR-141-5p				
miR-429	**9.1**	**27.0**	**11.8**	**4.8**
miR-96-5p	**11.7**	**25.6**	**20.0**	**5.0**

*Clusters miR-96/182/183, miR-141/200c, and miR-200a/200b/429 specify 10 miRNAs in male adult ARC. Eight miRNAs displayed MAX/MINs higher than the value of 8.3 (i.e., mean + 2 STD) in group C-C as well in groups C-HF, HF-C, and HF-HF (Bold numbers). miRNAs miR-200a-3p and miR-141-3p displayed MAX/MINs higher than the value of 8.3 in groups HF-C and/or HF-HF*.

In groups C-HF, HF-C, and HF-HF, more than 94% of miRNAs also displayed close MAX/MINs and CVs with 5.5, 5.6, and 1.3% of the miRNAs having MAX/MINs larger than the threshold value of 8.3, respectively. In these groups, miRNAs produced from clusters miR-96/182/183, miR-141/200c, miR-200a/200b/429 also appeared as hypervariable miRNAs (see Table 2). In groups C-HF, HF-C, and HF-HF as in group C-C, the expression of the 10 miRNAs expressed by these clusters co-varies in each profile (see Figure 5).

The extensive intra-group homogeneity of miRNA expression profiles of each group is an important feature for sound inter-group differential expression analyses.

### miRNA expression profiles are impacted by perinatal metabolic environments

We then compared miRNA expressions between groups C-C and C-HF, HF-C, or HF-HF (Supplemental Table S4). The 24 expression profiles were normalized together, *p*-values were calculated and corrected for false discovery rates (see section Materials and Methods). The padj-value of 1.0E-2 was retained as the first threshold significant value.

**Figure 6:**
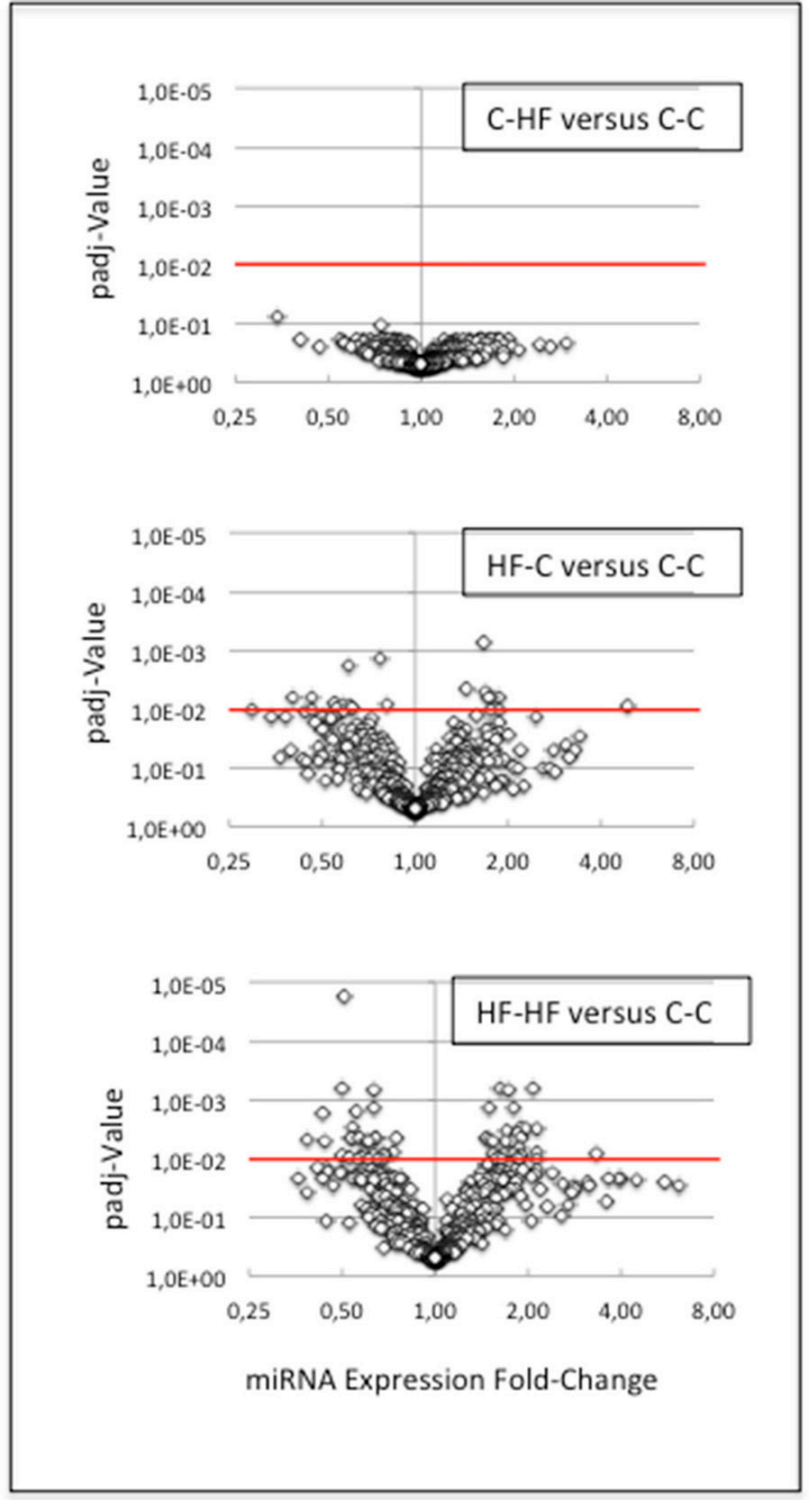
miRNA response to metabolic challenge. We compared miRNA expressions between groups C-C and C-HF, HF-C, or HF-HF. For each comparison, the volcano plot displays padj-values plotted against expression fold-changes. X- and Y-axes are drawn using log_2_ and log_10_ scales, respectively. Values higher than 1 on the X-axis denote up-regulated miRNAs in groups C-HF, HF-C, or HF-HF, and values lower than 1, down-regulated miRNAs. Red lines identify the threshold padj-value of 1.0E-2. **(Upper Plot)** C-HF vs. C-C comparison. All padj-values were higher than 7.0E-2. miRNAs did not display any expression difference. **(Middle Plot)** HF-C vs. C-C comparison. Eighteen miRNAs displayed significant difference of expression. **(Lower Plot)** HF-HF vs. C-C comparison. Sixty miRNAs displayed significant difference of expression. The expression of several miRNAs was impacted by the perinatal only, or perinatal and adult, unbalanced environment.

miRNAs did not display any expression difference between groups C-C and C-HF: all padj-values were higher than 0.07 (Figure 6).

In contrast, 18 out of 433 miRNAs (4%) displayed significant difference of expression between groups C-C and HF-C (padj-value = < 1.0E-2) (see Figure 6) (Table 3). Fold-changes of expression (FCEs) ranged from 0.4 to 2 except in the case of miR-3585-5p that displayed a FCE of 4.8. FCEs are expected to occur with similar levels and associated padj-values in cases where miRNAs are specified by tightly linked genes transcribed as one RNA unit. This is the case for 1 of the 18 miRNAs. miR-3585-5p and -547-3p maturate from the same precursor and miR-547-3p did display similar FCE (3.3) and padj-value (5.0E-2).

**Table 3:** miRNA and partner-miRNA expression in groups HF-C vs. C-C.

**miRNAs Differentially Expressed Between Groups C-C and HF-C**					
**miRNA**	**FCE**	**Padj-value**	**Partner-miRNA**	**FCE**	**Padj-value**
let-7i-5p	1.9	6.5E-03	let-7i-3p	1.5	3.1E-01
miR-107-3p	0.6	9.3E-03			
miR-1188-5p	0.4	7.0E-03			
miR-132-3p	1.7	5.1E-03	miR-132-5p	1.3	2.5E-01
miR-140-5p	1.7	6.5E-03	miR-140-3p	0.9	1.9E-01
miR-28-5p	1.7	6.9E-03	miR-28-3p	0.8	9.3E-02
miR-221-5p	1.9	1.0E-02	miR-221-3p	1.6	6.2E-02
miR-323-5p	0.5	6.5E-03	miR-323-3p	0.5	1.1E-02
miR-338-5p	1.5	4.2E-03	miR-338-3p	1.4	3.1E-01
miR-3585-5p	4.8	9.3E-03	miR-3585-3p	3.4	3.1E-02
miR-433-3p	0.6	1.0E-02	miR-433-5p	0.6	4.7E-02
miR-485-3p	0.6	1.8E-03	miR-485-5p	0.8	4.2E-02
miR-543-3p	0.8	6.9E-03	miR-543-5p	0.6	2.2E-02
miR-668	0.6	9.3E-03			
miR-673-5p	0.5	6.5E-03			
miR-708-5p	1.6	7.7E-04	miR-708-3p	0.9	3.4E-01
miR-770-3p	0.6	1.0E-02	miR-770-5p	0.6	1.2E-02
miR-92a-3p	0.8	1.8E-03			

*Eighteen miRNAs displayed expression differences with padj-values = < 1.0E-2 between groups C-C and HF-C. Ratios of HF-C to C-C expressions (Fold-Changes of Expression; FCEs) are expected to occur with similar levels and associated padj-values for the miRNA and second strand (Partner-miRNA) derived from the same RNA duplex precursor. Thirteen miRNAs were identified along with their Partner-miRNAs. Six Partner-miRNAs, i.e., miR-323-5p, −3585-5p, −433-3p, −485-3p, −543-5p, and −770-3p displayed close FCEs and padj-values = < 5.0E-2. Seven Partner-miRNAs (shaded Partner-miRNAs) displayed similar FCEs but padj-values >5.0E-2. miRNAs are listed by alphabetical order*.

miRNAs maturate from imperfect duplexes of some 22 basepairs, themselves arisen from RNA imperfect hairpins of some 70 nucleotides. miRNA maturation involves the incorporation of one duplex strand/miRNA into a RNA-induced silencing complex (RISC). For almost all duplexes, each strand can be incorporated within a RISC. Depending on the duplex, incorporation can display similar or highly different efficiencies between the two strands designed as miRNA-3p and miRNA-5p according to the hairpin arm from which they arise. We reasoned that FCEs of miRNA-3p and miRNA-5p derived from the same duplex are expected to vary similarly for all or almost all duplexes and used this feature to validate FCEs observed between groups C-C and HF-C.

**Figure 7:**
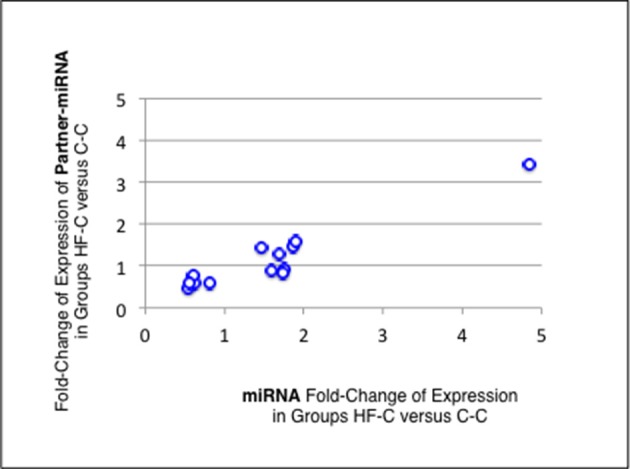
miRNA and partner-miRNA differential expression in groups HF-C vs. C-C. Thirteen miRNAs differentially expressed between groups C-C and HF-C maturated in parallel with the second strand of their RNA duplex precursor (Partner-miRNA). Ratios of HF-C to C-C expressions (Fold-Change of Expression; FCE) are expected to occur with similar levels for a miRNA and its Partner-miRNA. FCEs of miRNA-Partners were plotted against FCEs of miRNAs. Both FCEs displayed high correlation (*R*^2^ = 0.92).

**Figure 8:**
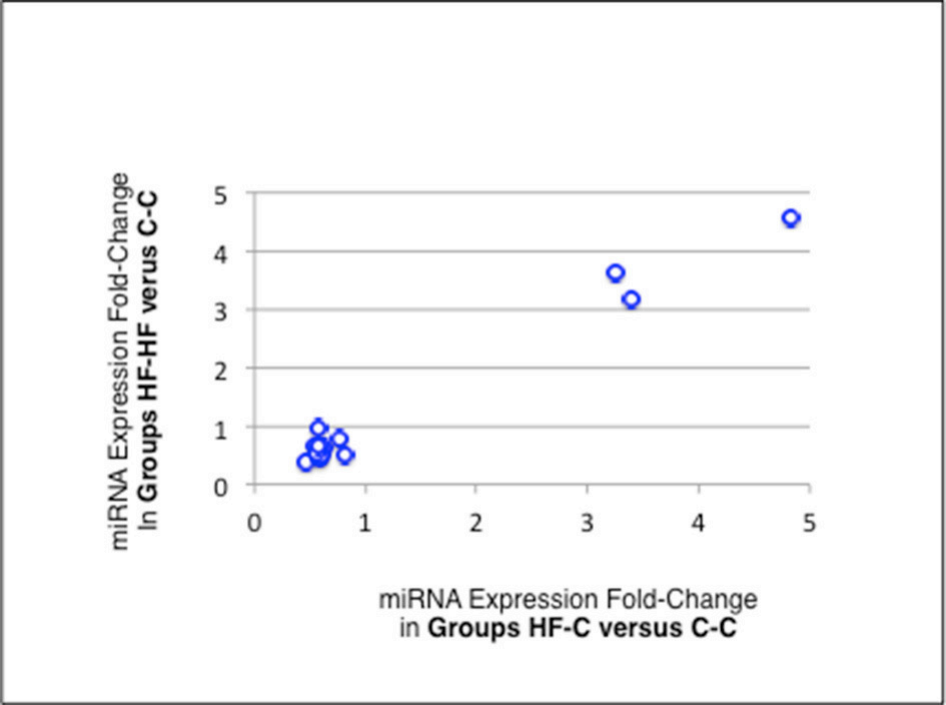
miRNA differential expression in groups HF-C or HF-HF vs. C-C. miRNAs and Partner-miRNAs specified by the genes miR-323,−3585,−433,−485, and−770 as well as the miRNA miR-547-3p appeared significantly impacted by the sole perinatal unbalanced environment (padj-value < 5.2E-02; see Table 3). For each of these miRNAs, the Fold-Change of Expression (FCE) in Groups HF-HF vs. C-C was plotted against the FCE in Groups HF-C vs. C-C. Both FCEs displayed high correlation (*R*^2^ = 0.98) indicating that the 11 miRNAs defined a subset of miRNAs repeatedly impacted by the perinatal unbalanced environment.

Thirteen of the eighteen miRNAs differentially expressed between groups C-C and HF-C maturated in parallel with the second duplex strand (hereafter named Partner-miRNA) (see Table 3). Those 13 miRNAs and Partner-miRNAs displayed highly correlated FCEs (*R*^2^ = 0.92) (Figure 7). In six cases, i.e., miR-323-3p, −3585-3p, −433-5p, −485-5p, −543-3p, and −770-5p, Partner-miRNAs, 323-5p, −3585-5p, −433-3p, −485-3p, −543-5p, and −770-3p, also displayed very close padj-values (<5.0E-2). Therefore 13 miRNAs, i.e., the two strands specified by genes miR-323, −3585, −433, −485, −543, and −770 as well as the miR-547-3p strand appeared impacted by the perinatal unbalanced environment. All these miRNAs except miR-543-5p also displayed significant changes of expression (padj-value =< 5.2E-02) between groups C-C and HF-HF (Table 4). miRNA FCEs between profiles of groups C-C and HF-C highly correlated with miRNA FCEs between profiles of groups C-C and HF-HF (*R*^2^ = 0.98) (Figure 8). Based on these criteria, 11 miRNAs, i.e., the two strands specified by the genes miR-323, −3585, −433, −485, and −770 as well as the 3p strand defined by the gene miR-547 defined a subset of miRNAs repeatedly impacted by the perinatal unbalanced environment.

**Table 4:** miRNA expression in groups HF-C and HF-HF.

	**miRNAs Differentially Expressed Between**			
**miRNA**	**Groups C-C and HF-C**		**Groups C-C and HF-HF**	
miRNA	FCE	Padj-value	FCE	Padj-value
miR-323-3p	0.5	1.1E-02	0.4	5.3E-03
miR-323-5p	0.5	6.5E-03	0.7	5.3E-03
miR-3585-3p	3.4	3.1E-02	3.2	2.4E-02
miR-3585-5p	4.8	9.3E-03	4.6	2.3E-02
miR-433-3p	0.6	1.0E-02	0.6	2.0E-03
miR-433-5p	0.6	4.7E-02	0.5	1.7E-02
miR-485-3p	0.6	1.8E-03	0.5	5.1E-04
miR-485-5p	0.8	4.2E-02	0.8	2.2E-02
miR-543-3p	0.8	6.9E-03	0.5	1.7E-05
miR-543-5p	0.6	2.2E-02	1.0	4.2E-01
miR-547-3p	3.3	5.0E-02	3.6	5.2E-02
miR-770-3p	0.6	1.0E-02	0.5	5.3E-03
miR-770-5p	0.6	1.2E-02	0.7	1.1E-02

*Thirteen miRNAs, i.e., the two strands specified by the genes miR-323, −3585, −433, −485, −543, and −770 as well as the miR-547-3p strand were differently expressed in groups HF-C vs. CC (see Table 3). Remarkably all these miRNAs except miR-543-5p (shaded miRNA) also displayed significant changes of expression (padj-value = < 5.2E-02) between groups C-C and HF-HF, thus defining a subset of miRNAs repeatedly impacted by the perinatal unbalanced environment. miRNAs are listed by alphabetical order*.

**Figure 9:**
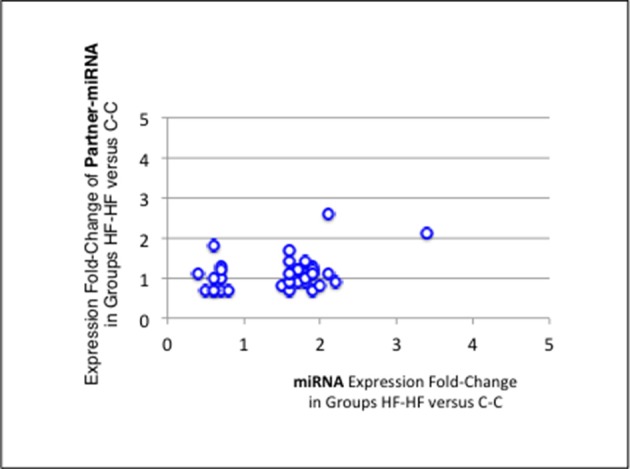
miRNA and partner-miRNA differential expression in groups HF-HF vs. C-C. Thirty-four miRNAs specifically differentially expressed between groups C-C and HF-HF maturated in parallel with the second strand of their RNA duplex precursor (Partner-miRNA). Ratios of expressions (Fold-Change of Expression; FCE) of Partner-miRNAs were plotted against FCEs of miRNAs. Both FCEs displayed low correlation (*R*^2^ = 0.15).

Finally, when comparing profiles of groups C-C and HF-HF, 49 additional miRNAs displayed significant difference of expression (padj-value = < 1.0E-2) (Table 5). Thirty-four of these miRNAs maturated in parallel with the second duplex strand (see Table 5). Those 34 miRNAs and Partner-miRNAs displayed low correlated FCEs (R^2^ = 0.15) (Figure 9). In nine cases, i.e., miR-127-3p, −139-3p, −140-5p, 145-5p, 27a-3p, 29a-3p, −344-b1-3p, 369-5p, and −409a-5p, miRNAs and Partner-miRNAs displayed close FCEs and padj-values (= < 5.0E-2). For miR-145-5p, −27a-3p, −29a-3p, −344b-3p, −369-5p, and −409a-5p that lay within miR gene clusters potentially transcribed as single RNA units, we noted that associated miRNAs displayed similar FCEs and padj-values.

**Table 5:** miRNA and partner-miRNA expression in groups HF-HF vs. C-C.

**miRNAs Differentially Expressed Between Groups C-C and HF-HF**					
**miRNA**	**FCE**	**Padj-value**	**Partner miRNA**	**FCE**	**Padj-value**
let-7a-1-3p	1.9	4.0E-03	let-7a-5p	0.7	6.5E-02
let-7i-5p	1.6	6.4E-03	let-7i-3p	1.4	5.6E-02
miR-1188-5p	0.4	5.7E-03			
miR-1224	0.5	5.1E-03			
miR-127-3p	0.7	9.2E-03	miR-127-5p	0.7	2.3E-02
miR-132-3p	1.9	6.6E-03	miR-132-5p	1.1	2.8E-01
miR-134-3p	1.8	8.1E-03	miR-134-5p	0.9	6.3E-02
miR-139-3p	0.6	5.7E-03	miR-139-5p	0.7	2.3E-02
miR-140-5p	2.1	3.0E-04	miR-140-3p	1.1	3.3E-02
miR-145-5p	3.4	8.1E-03	miR-145-3p	2.1	1.8E-02
miR-149-5p	0.6	5.1E-03			
miR-153-3p	1.9	9.7E-03	miR-153-5p	1.2	1.8E-01
miR-154-5p	1.5	4.9E-03	miR-154-3p	0.8	6.5E-02
miR-1843b-3p	0.6	9.1E-03			
miR-185-5p	1.5	4.5E-03			
miR-192-5p	1.7	2.9E-03			
miR-212-3p	1.9	3.1E-03	miR-212-5p	1.3	5.1E-02
miR-23a-3p	2.0	3.0E-03	miR-23a-5p	0.8	2.1E-01
miR-25-3p	1.6	5.7E-03	miR-25-5p	0.7	1.5E-02
miR-27a-3p	2.1	1.0E-02	miR-27a-5p	2.6	2.7E-02
miR-27b-3p	1.8	5.7E-03	miR-27b-5p	1.4	8.1E-02
miR-28-5p	1.7	5.1E-03	miR-28-3p	0.9	3.3E-01
miR-298-3p	0.4	2.0E-02	miR-298-5p	1.1	2.7E-01
miR-29a-3p	1.6	9.7E-03	miR-29a-5p	1.7	2.1E-02
miR-3068-5p	1.8	8.1E-04	miR-3068-3p	1.0	4.8E-01
miR-329-3p	0.7	9.7E-03	miR-329-5p	1.0	4.8E-01
miR-335	1.5	2.0E-03			
miR-344b-1-3p	0.5	9.7E-03	miR-344b-5p	0.7	2.1E-02
miR-34a-5p	2.1	2.9E-02			
miR-34c-5p	1.9	2.9E-03	miR-34c-3p	1.2	2.2E-01
miR-369-5p	0.8	5.3E-03	miR-369-3p	0.7	4.4E-02
miR-376b-3p	0.6	9.7E-03	miR-376b-5p	1.0	4.4E-01
miR-376c-3p	1.8	7.0E-04			
miR-409a-5p	0.6	9.9E-03	miR-409a-3p	0.7	2.5E-02
miR-410-3p	0.5	9.5E-03			
miR-434-3p	0.7	9.7E-03	miR-434-5p	1.3	5.9E-02
miR-487b-3p	0.6	5.3E-03			
miR-493-5p	0.6	9.7E-03	miR-493-3p	1.8	1.7E-02
miR-495	0.6	2.0E-03			
miR-504	0.6	6.7E-04			
miR-505-5p	0.7	1.0E-02	miR-505-3p	1.2	4.0E-02
miR-539-3p	2.2	7.7E-03	miR-539-5p	0.9	1.5E-01
miR-652-5p	1.9	7.8E-03	miR-652-3p	1.1	2.6E-01
miR-668	0.6	5.8E-03			
miR-708-5p	1.6	3.6E-04	miR-708-3p	0.9	3.1E-01
miR-760-3p	0.5	2.9E-03			
miR-7a-1-3p	1.7	8.1E-03	miR-7a-5p	1.2	2.0E-01
miR-7a-2-3p	1.7	1.0E-02	miR-7a-5p	1.2	2.0E-01
miR-872-5p	1.6	9.7E-03	miR-872-3p	1.1	2.7E-01

*Forty-nine additional miRNAs displayed expression differences with padj-values = < 1.0E-2 between groups C-C and HF-HF. Thirty-four miRNAs were identified along with their Partner-miRNAs. Nine Partner-miRNAs displayed close FCEs and padj-values = < 5.0E-2. Twenty-five Partner-miRNAs (shaded Partner-miRNAs) displayed different FCEs and/or padj-values >5.0E-2. miRNAs are listed by alphabetical order*.

Comparisons of the expression profiles of groups C-C, HF-C, C-HF, and HF-HF showed that the expression of 29 miRNAs/Partner-miRNAs of the ARC of adult rats was impacted by the perinatal unbalanced environment. For 18 miRNAs/Partner-miRNAs, this impact relied on consecutive perinatal and adult exposures to an unbalanced metabolic environment.

## Discussion

Here, we extensively characterized miRNA populations in ARC of adult male rats of four metabolic statuses resulting from early and/or adult, balanced and/or unbalanced metabolic environments. We show that the ARC of adults holds a population of about 400 miRNA species that is as complex as the one of the first weeks of life (Doubi-Kadmiri et al., 2016). More than 94% of the miRNAs displayed homogeneous expression in the four groups C-C, C-HF, HF-C, and HF-HF. For 29 of those miRNAs, the perinatal environment impacts the expression.

### The specific case of clusters miR-96/182/183, miR-141/200c, and miR-200a/200b/429

In each metabolic status, a dozen of miRNAs exhibited hyper-variable expression. Among those miRNAs were all the 10 miRNAs produced from miR gene clusters miR-96/182/183, miR-141/200c, and miR-200a/200b/429. The 10 miRNAs co-varied in each profile demonstrating that the expression variability was due to true biological variations of expression rather than to experimental changes. Co-variation of miRNA expression also demonstrated that the transcription and maturation of clusters miR-96/182/183, miR-141/200c, and miR-200a/200b/429 are co-regulated in ARC.

miRNAs interact with targeted mRNAs primarily through nucleotides 2–7 (the seed region). In some cases, miRNA:mRNA interactions involve additional nucleotides. miRNA-5ps specified by the three clusters displayed low sequence identity and have four seed regions. miRNA-3ps specified by the three clusters displayed extensive sequence identity all along although the seed region of miR-141-3p and −200a-3p differs by one nucleotide at position 4 with that of miR-200b-3p, −200c-3p, and −429-3p. Large differences in the level of the seed regions of the miRNAs specified by the three clusters are expected to promote different levels of proteins encoded by targeted mRNAs. This may underlie differences in adult functions of ARC and, later, in individual susceptibilities to aging effects.

miRNAs specified by clusters miR-96/182/183, miR-141/200c, and miR-200a/200b/429 have been identified as miRNAs of functional importance in sensory organs. Punctual mutation or deletion in cluster miR-96/182/183 produces ear or retina disorders while loss of function of the miR-200 family leads to defects in the terminal differentiation of olfactory precursors (Choi et al., 2008; Kuhn et al., 2011; Lumayag et al., 2013). Whether expression of those miRNAs in sensory organs also varies between individuals deserves investigation.

### Adult unbalanced metabolic environment has no impact on miRNA expression in ARC

miRNAs of the ARC of rats exposed to the unbalanced metabolic environment at the sole adulthood period display similar expression as those of rats exposed to the balanced environment. This absence of effect is observed although rats exposed to the unbalanced environment had two- and three-fold more circulating leptin and insulin, respectively, than rats exposed to the balanced environment. Levels of circulating fat are also probably highly different. Indeed, adult rats subjected to similar balanced or unbalanced metabolic environment have been reported to exhibit a seven-fold difference in plasma triglycerides (Auberval et al., 2014). This demonstrates that the transcription of the miRNA precursors of adult ARC is not affected by differences of such orders in circulating fat, leptin and insulin, even though leptin and insulin signaling pathways are known to include the activation of several transcription factors. Our work also suggests that miRNAs of adult ARC are not crucial for setting up carbohydrate and lipid peripheral metabolisms appropriate to balanced or unbalanced metabolic environment.

### Early unbalanced metabolic environment affects miRNA expression in adult ARC

**Figure 10:**
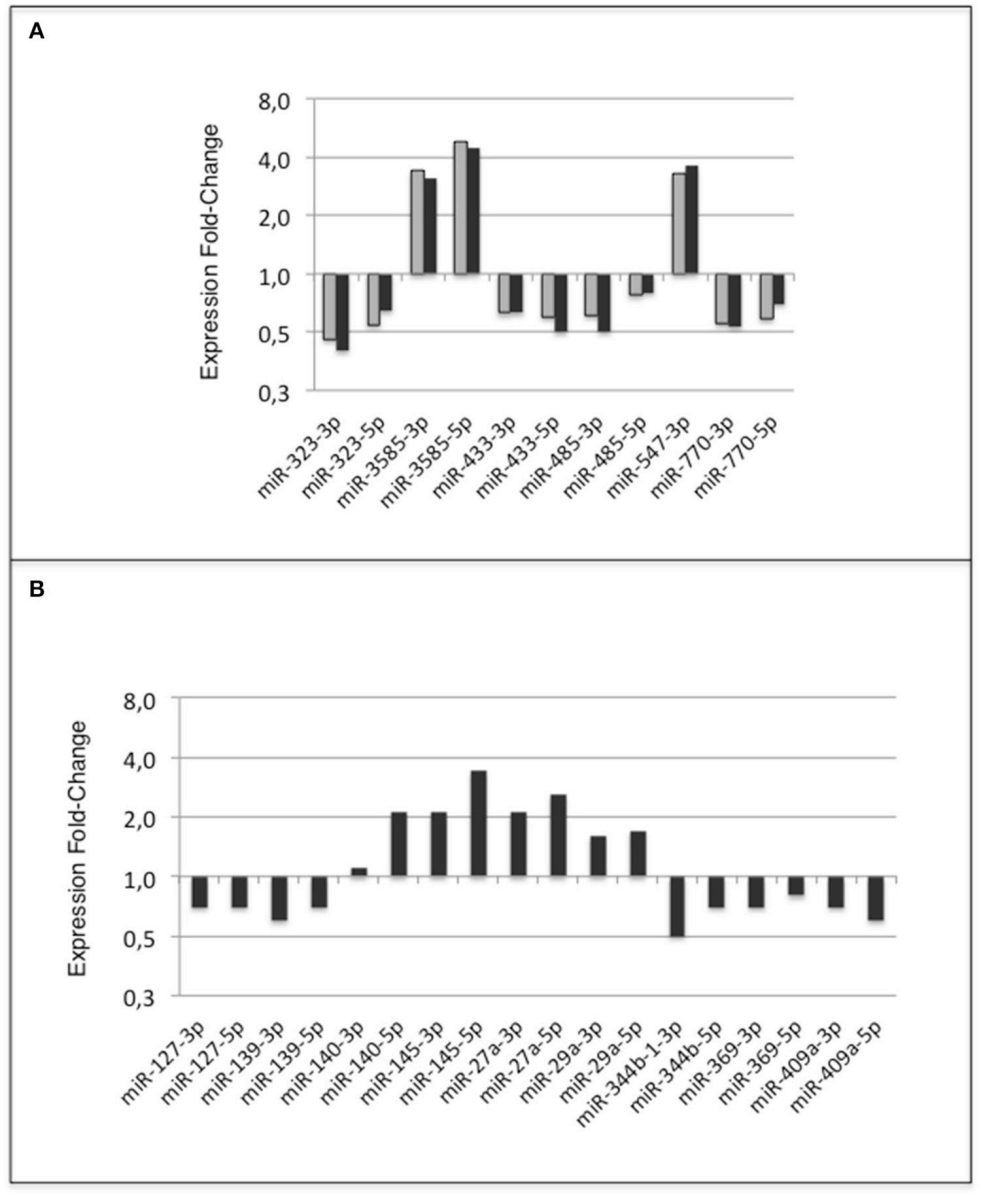
miRNA response of adult ARC to metabolic environment. Summary **(A)** miRNA response to early metabolic challenge. Fold-Changes of Expression (FCE) were observed in Groups HF-C vs. C-C (gray bars) and Groups HF-HF vs. C-C (black bars); **(B)** Additional miRNA response to consecutive adult metabolic challenge observed in Groups HF-HF relatively to C-C. FCEs are shown using a log_2_ scale.

Eleven miRNAs of ARC of adult rats that have been exposed to the unbalanced metabolic environment during the perinatal period regularly displayed expression changes (Figure 10A). The five miRNAs miR-323-3p, −3585-3p, −433-5p, −485-5p, and −770-5p, the five Partner-miRNAs, 323-5p, −3585-5p, −433-3p, −485-3p, and−770-3p and the closely linked miRNA miR-547-3p defined a subset of miRNAs repeatedly impacted by the perinatal unbalanced environment.

Additionally, 18 miRNAs displayed expression differences in ARC of rats consecutively exposed to an unbalanced environment during the perinatal and adult periods, i.e., the nine miRNAs miRs-127-3p, −139-3p, −140-5p, −145-5p, −27a-3p, −29a-3p, −344b-3p, −369-5p, and −409a-5p, and nine Partner-miRNAs miRs-127-5p, −139-5p, −140-3p, −145-3p, −27a-5p, −29a-5p, −344b-5p, −369-3p, and −409a-3p (Figure 10B). Consecutive exposure to an unbalanced environment during the perinatal and adult periods led to slightly higher levels of circulating glucose (see Figure 3). Glucose controls the expression of a glucose-responsive transcription factor (ChREBP) that regulates fatty acid synthesis and glycolysis in liver and adipose tissue. The role of ChREBP in the hypothalamus is still unknown.

We previously reported a significant (*p* < 5.0E-2) expression difference of miR-125a-3p, −200a-3p, and −409-5p in the hypothalamus of male adult rats fed a HF-diet for their last 4 weeks of life (this study used the same C- and HF-diets than the ones used here) in relation to differences in their postnatal environment (Benoit et al., 2013). Rats that had received a daily injection of a pegylated rat leptin antagonist (pRLA) from day 2 to day 13, displayed a slight enhancement of expression of the three miRNAs at the age of 4 months when compared to rats injected saline (FCEs ranged from 1.2 to 1.5).

In our study, miR-125a-3p displayed similar expression between the four groups (padj-values > 1.2E-01 in all comparisons; see Supplemental Table S5). In contrast, miR-200a-3p and −409-5p displayed trends to expression differences (padj-values > 1.0E-2 but < 5.0E-2) between groups C-C or C-HF on one hand and groups HF-C or HF-HF on the other hand. Along with miR-200a-3p, the miRNAs miR-200a-5p, −200b-3p, −200b-5p, and −429 that are specified by the same gene cluster displayed similar FCEs (ranging from 2.0 to 3.4) and padj-values. Of note, the miRNAs miR-429, −182, −183-3p, −183-5p, and −96-5p which expression co-varied with that of with miR-200a-3p in all expression profiles (see above) also displayed similar FCEs (ranging from 2.8 to 6.2) and padj-values (>1.0E-2 but <5.0E-2). Similarly, along with miR-409a-5p, the miRNAs miR-409a-3p, −412-3p, and −412-5p that are specified by the same gene cluster displayed similar FCEs (ranging from 0.3 to 0.7) and padj-values when comparing group HF-C or HF-HF vs. group C-C.

Unbalanced perinatal metabolic environment and daily postnatal injection of pRLA have therefore different consequences on miR-125a-3p (constant expression vs. up-regulation, respectively) and −409-5p expression (down- vs. up-regulation, respectively). This may be due to differences in the experimental protocol itself, origin of miRNAs (the ARC vs. the whole hypothalamus) or age of animal (7 vs. 4 months). In contrast, miR-200a-3p expression, and potentially the expression of all miRNAs specified by the three clusters miR-96/182/183, miR-141/200c, and miR-200a/200b/429, tend to be up-regulated with similar FCEs in both experimental conditions.

We also reported up-regulation of miR-200a-3p,−200b-3p, and−409 in the hypothalamus of Lep°^b^/Lep°^b^ mice deficient in leptin production when compared to Lep°^b^/Lep^+^ or Lep^+^/Lep^+^ mice (Crépin et al., 2014). In this study we also showed that daily intra-peritoneal injections of leptin significantly reduced hypothalamic expression of the three miRNAs in Lep°^b^/Lep°^b^ but not in Lep°^b^/Lep^+^ mice. On the other hand, intra-cerebroventricular (ICV) infusion of anti-miR-200a-3p to Lep°^b^/Lep°^b^ mice restored the hypothalamic expression of *Irs-2* and *Lepr*, and up-regulated the hypothalamic expression of *Zfpm2, Insr, Pomc*, and *Npy. Pomc* and miR-200a-3p expressions did not display any correlation or anti-correlation in groups C-C, C-HF, HF-C, and HF-HF of our study (*R*^2^ = 0.09). The increase of *Pomc* expression in infused mouse hypothalami may be due to the use of over-physiological quantities of anti-miR-200a-3p. Alternatively *Pomc* expression may be under different controls in mouse and rat.

Altogether our new and previous findings point to a trend to the long-term overexpression of clusters miR-96/182/183, miR-141/200c, and miR-200a/200b/429 in the ARC of adult rats and mice having experienced low levels of leptin/leptin signaling during the perinatal period.

Long-term consumption of a HFD-diet has been shown to induce microglia activation in hypothalamus of rats and mice (Thaler et al., 2012). Indeed the expression of several pro-inflammatory genes was increased by approximately 50% in the hypothalamus of adult male rats subjected to a HFD-diet of 4–20 weeks relatively to control rats fed a standard diet. Changes of microglial accumulation and cell size were limited to the ARC/Median eminence area. miR-146a and miR-155 appeared to play specific roles in brain inflammatory responses (Cardoso et al., 2016). miR-146a and miR-155 displayed similar expression between groups C-C and C-HF of our study (see Supplemental Table 4; padj-values > 3.0E-01). This suggests that adult HF-diet *per se*, at least the one we used, promotes low or no brain inflammatory response. miR-146a and −155 however displayed trends of up-regulation in groups HF-C and HF-HF when compared to group C-C (FCEs: 1.7–2.0; padj-values < 7.0E-02). The perinatal HF-diet may therefore induce long-term inflammation of ARC.

## Conclusion

miRNAs participate in the control of many signaling pathways, many of which are involved in epigenetics regulations. Our findings reveal that miRNA expression in ARC at adulthood can change as a consequence of the quality of the early metabolic environment. Which among high fat, high carbohydrate, and/or moderate protein content in early life is responsible for miRNA differential expression in adult ARC needs to be determined. miRNA expression changes were modest with fold-changes lower than two except for a few miRNAs. *In vivo* identification of the mRNAs targeted by the differentially expressed miRNAs and characterization of the impact of miRNA expression differences on the synthesis of the mRNA-encoded proteins will determine whether and how these changes alter ARC functions.

## Accession codes

Raw data files have been submitted at the SRA database (NCBI) under the study accession number SRP058705.

## Author contributions

CB, SD-K, C-MV, MT, AB-T, and LA: Conceived and designed the experiments; SD-K, CB, DC, GP, LR, C-MV, MT, AB-T, and LA: Performed the experiments; XB: Developed the algorithms; SD-K, CB, XB, AB-T, and LA: Analyzed the data; AB-T and LA: Wrote the paper. All authors reviewed the manuscript.

### Conflict of interest statement

The authors declare that the research was conducted in the absence of any commercial or financial relationships that could be construed as a potential conflict of interest.

## References

[B1] Ainge H. Thompson C. Ozanne S. E. Rooney K. B. (2011). A systematic review on animal models of maternal high fat feeding and offspring glycaemic control. Int. J. Obes. 35, 325–335. 10.1038/ijo.2010.149 20680016

[B2] Amar L. Benoit C. Beaumont G. Vacher C. M. Crepin D. Taouis M.. (2012). MicroRNA expression profiling of hypothalamic arcuate and paraventricular nuclei from single rats using Illumina sequencing technology. J. Neurosci. Methods. 209, 134–143. 10.1016/j.jneumeth.2012.05.033 22687940

[B3] Anders S. Huber W. (2010). Differential expression analysis for sequence count data. Genome Biol. 11:R106. 10.1186/gb-2010-11-10-r106 20979621 PMC3218662

[B4] Auberval N. Dal S. Bietiger W. Pinget M. Jeandidier N. Maillard-Pedracini E.. (2014). Metabolic and oxidative stress markers in Wistar rats after 2 months on a high-fat diet. Diabetol. Metab. Syndr. 6:130. 10.1186/1758-5996-6-130 25960774 PMC4424531

[B5] Baroin-Tourancheau A. Benigni X. Benoit C. Doubi-Kadmiri S. Vacher C. M. Taouis M.. (2014). Keys for microRNA expression profiling of single rat hypothalamic nuclei and multiplex sequencing strategies. Exp. Physiol. 99, 72–77. 10.1113/expphysiol.2013.072546 24243838

[B6] Baroin-Tourancheau A. Benigni X. Doubi-Kadmiri S. Taouis M. Amar L. (2016). Lessons from microRNA sequencing using Illumina technology. Adv. Biol. Biotechnol. 7, 319–328. 10.4236/abb.2016.77030

[B7] Bartel D. P. (2009). MicroRNAs: target recognition and regulatory functions. Cell 13, 215–233. 10.1016/j.cell.2009.01.002 PMC3794896 19167326

[B8] Benjamini Y. Hochberg Y. (1995). Controlling the False Discovery Rate: a practical and powerful approach to multiple testing. J. R. Stat. Soc. Ser. B 57, 289–300. 10.2307/2346101

[B9] Benoit C. Ould-Hamouda H. Crepin D. Gertler A. Amar L. Taouis M. (2013). Early leptin blockade predisposes fat-fed rats to overweight and modifies hypothalamic microRNAs. J. Endocrinol. 218, 35–47. 10.1530/JOE-12-0561 23576026

[B10] Cardoso A. L. Guedes J. R. de Lima M. C. (2016). Role of microRNAs in the regulation of innate immune cells under neuroinflammatory conditions. Curr. Opin. Pharmacol. 26, 1–9. 10.1016/j.coph.2015.09.001 26410391

[B11] Choi P. S. Zakhary L. Choi W. Y. Caron S. Alvarez-Saavedra E. Miska E. A.. (2008). Members of the miRNA-200 family regulate olfactory neurogenesis. Neuron 57, 41–55. 10.1016/j.neuron.2007.11.018 18184563 PMC2204047

[B12] Crépin D. Benomar Y. Riffault L. Amine H. Gertler A. Taouis M. (2014). The over-expression of miR-200a in the hypothalamus of ob/ob mice is linked to leptin and insulin signaling impairment. Mol. Cell. Endocrinol. 384, 1–11. 10.1016/j.mce.2013.12.016 24394757

[B13] Doubi-Kadmiri S. Benoit C. Benigni X. Beaumont G. Vacher C. M. Taouis M.. (2016). Substantial and robust changes in microRNA transcriptome support postnatal development of the hypothalamus in rat. Sci. Rep. 6:24896. 10.1038/srep24896 27118433 PMC4847009

[B14] Griffiths-Jones S. Saini H. K. van Dongen S. Enright A. J. (2008). miRBase: tools for microRNA genomics. Nucleic Acids Res. 36, D154–D158. 10.1093/nar/gkm952 17991681 PMC2238936

[B15] Ha M. Kim V. N. (2014). Regulation of microRNA biogenesis. Nat. Rev. Mol. Cell Biol. 8, 509–524. 10.1038/nrm3838 25027649

[B16] Hanson M. A. Gluckman P. D. (2014). Early developmental conditioning of later health and disease: physiology or pathophysiology? Physiol. Rev. 94, 1027–1076. 10.1152/physrev.00029.2013 25287859 PMC4187033

[B17] Kuhn S. Johnson S. L. Furness D. N. Chen J. Ingham N. Hilton J. M.. (2011). miR-96 regulates the progression of differentiation in mammalian cochlear inner and outer hair cells. Proc. Natl. Acad. Sci. U.S.A. 108, 2355–2360. 10.1073/pnas.1016646108 21245307 PMC3038748

[B18] Lumayag S. Haldin C. E. Corbett N. J. Wahlin K. J. Cowan C. Turturro S.. (2013). Inactivation of the microRNA-183/96/182 cluster results in syndromic retinal degeneration. Proc. Natl. Acad. Sci. U.S.A. 110, E507–E516. 10.1073/pnas.1212655110 23341629 PMC3568372

[B19] Rodríguez E. M. Blázquez J. L. Guerra M. (2010). The design of barriers in the hypothalamus allows the median eminence and the arcuate nucleus to enjoy private milieus: the former opens to the portal blood and the latter to the cerebrospinal fluid. Peptides 31, 757–776. 10.1016/j.peptides.2010.01.003 20093161

[B20] Rueda A. Barturen G. Lebron R. Gomez-Martin C. Aldanza A. Oliver J. L. (2015). sRNAtoolbox: an integrated collection of small RNA research tool. Nucl. Acids Res. 43, W467–W473. 10.1093/nar/gkv555 26019179 PMC4489306

[B21] Sobrino Crespo C. Perianes Cachero A. Puebla Jiménez L. Barrios V. Arilla Ferreiro E. (2017). Peptides and food intake. Front. Endocrinol. 5:58. 10.3389/fendo.2014.00058 24795698 PMC4005944

[B22] Thaler J. P. Yi C. X. Schur E. A. Guyenet S. J. Hwang B. H. Dietrich M. O. (2012). Obesity is associated with hypothalamic injury in rodents and humans. J. Clin. Invest. 22, 153–162. 10.1172/JCI59660 PMC3248304 22201683

[B23] Timper K. Brüning J. C. (2017). Hypothalamic circuits regulating appetite and energy homeostasis: pathways to obesity. Dis. Model. Mech. 10, 679–689. 10.1242/dmm.026609 28592656 PMC5483000

[B24] Vienberg S. Geiger J. Madsen S. Dalgaard L. T. (2017). MicroRNAs in metabolism. Acta Physiol. 219, 346–361. 10.1111/apha.12681 27009502 PMC5297868

